# An Impertinent Suggestion

**Published:** 1916-08

**Authors:** 


### An Impertinent Suggestion.

The United States Public Health Service sends out some bulletins that contain suggestions that would start the people thinking could they only see them or hear of them. Here is one: "Go camping for your health and then place your toilet so that it drains into your water supply?" The question mark at the end of the sentence is hardly necessary to indicate the ironical character of the advice. Did you ever notice how many people do that very thing? Their health, they think, demands that they should go camping to live in the open air, which is a very proper thing to do. They pitch their tents alongside some stream, by the side of which, and close by, others are also likely camping. The first thing is to go in swimming, a sport in which all the family, including the dog, joins. Other people up and down stream are also in swimming. Some are sick, else they would not be out camping; some may even have loathsome diseases. Often cattle may be found standing all the day long in the stream to cool themselves and keep off the flies. Temporary toilets are arranged up and down the watershed of the stream. The water supply for the camp comes either from the stream or from a nearby well filled by seepage from the stream. Flies, mosquitoes and other insects abound, feeding at will on the filth from the camps, from the cows, horses, and dogs, and on the campers. Even where the water from the stream is not used for drinking or for cooking the meals it is used for indiscriminate bathing, and often where the quantity is so small or the current so slow that the swimming hole may easily scatter infection. There isn't much wonder that it takes a long while for some people to recover from the bad effects of a camping trip.

